# New Mode of Dilating Strictures of the Urethra

**Published:** 1849-02

**Authors:** 


*New mode of dilating Strictures of the Urethra.*—M. Amussat, in a case of stricture which resisted all treatment, and beyond which ordinary instruments could not be passed, finally succeeded in introducing a very fine bougie of half a millimeter, (the millimeter is equal to 1-26th of an inch English,) and, using this as a conductor, on the following days introduced alongside of this successively several others, to the number of six. Between these urine passed. They were left in for several days, being occasionally withdrawn and re-introduced in a bunch, passing as easily as a single bougie of the same size would. The stricture was now readily dilated with ordinary instruments and the cure rapidly effected. The advantage of this method is, that when once we can introduce an instrument, however small, there is no liability to failure in introducing the bougie a second time if once withdrawn, or in attempting to pass a larger one. Whatever is gained is maintained, and the first introduced serves as a guide to other instruments of the same size. The dilatation can thus be readily accomplished, and the urine passing between the small bougies, they can be retained several days without inconvenience.—*Ibid from Ibid.*

